# Evaluating Soil Moisture Status Using an e-Nose

**DOI:** 10.3390/s16060886

**Published:** 2016-06-22

**Authors:** Andrzej Bieganowski, Katarzyna Jaromin-Glen, Łukasz Guz, Grzegorz Łagód, Grzegorz Jozefaciuk, Wojciech Franus, Zbigniew Suchorab, Henryk Sobczuk

**Affiliations:** 1 Institute of Agrophysics, Polish Academy of Sciences, Doswiadczalna 4 Str., Lublin 20-290, Poland; a.bieganowski@ipan.lublin.pl (A.B.); g.jozefaciuk@ipan.lublin.pl (G.J); 2 Faculty of Environmental Engineering, Lublin University of Technology, Nadbystrzycka 40B Str., Lublin 20-618, Poland; l.guz@pollub.pl (Ł.G.); g.lagod@pollub.pl (G.Ł.); z.suchorab@pollub.pl (Z.S.); h.sobczuk@pollub.pl (H.S.); 3 Faculty of Civil Engineering and Architecture, Lublin University of Technology, Nadbystrzycka 40 Str., Lublin 20-618, Poland; w.franus@pollub.pl

**Keywords:** e-nose, humidity, metrology, smell, soil, water

## Abstract

The possibility of distinguishing different soil moisture levels by electronic nose (e-nose) was studied. Ten arable soils of various types were investigated. The measurements were performed for air-dry (AD) soils stored for one year, then moistened to field water capacity and finally dried within a period of 180 days. The volatile fingerprints changed during the course of drying. At the end of the drying cycle, the fingerprints were similar to those of the initial AD soils. Principal component analysis (PCA) and artificial neural network (ANN) analysis showed that e-nose results can be used to distinguish soil moisture. It was also shown that different soils can give different e-nose signals at the same moistures.

## 1. Introduction

Techniques of soil moisture estimation can be divided into direct and indirect ones. All of these methods have their own advantages and disadvantages. The gravimetric method (weighing the moist sample and the same sample after drying for 24 h at 105 °C) is a direct and non-questioned standard for water content determination in soil analysis; however, it is a laboratory-based test. An extension to field conditions is provided by indirect measurement using soil tensiometers or time-domain reflectometry (TDR) meters [1,2]. Tensiometers, equipped with a porous ceramic membrane sensor, directly measure the soil water potential (pF). This equipment has some severe limitations as its sensor has to be installed permanently in the same place in the soil, while the equilibrium time may be very long, thereby preventing the recording of faster changes in soil moisture. The TDR method, requires highly sophisticated and very expensive electronics, is based on the measurement of the apparent dielectric permittivity of the soil medium [3,4]. The TDR meters work properly for sandy and loamy soils, while individual calibration is needed for highly organic and/or clay-rich soils. A TDR meter uses rather long stainless steel wires as sensors, which are pushed into the soil [5,6]. This may cause sensor damage or changes in its geometry, severely limiting the applicability of TDR meters in on-the-go measuring systems. From this point of view a challenging problem concerns the application of the e-nose for estimating the soil moisture level. The cheap e-nose device, which provides a rather fast response and requires no direct contact with the soil, may overcome most limitations of the above methods. Moreover, it can be particularly useful for fast measurements, in which the direct contact of the sensor with a soil should be avoided. The e-nose moisture estimations, therefore, may be particularly useful for in-field measurements, including on-the-go systems, which are needed in precision agriculture or small-scale hydrology. The e-nose may also constitute a more advanced tool supporting the so-called practical intuition of soil scientists in estimation of soil types.

The e-nose imitates the human sense of smell; it is not an objective substance detector. All e-nose devices consist of the array of a few various gas sensors [7]. The use of many different sensors gives the ability to detect various gas components. A combination of signals from all sensors in the array characterizes the particular gas sample. This is comparable to fingerprints in dactyloscopy, because the probability of the same combination being formed for two different samples is very low; that is why the combination of these signals is popularly called a “gas fingerprint” [8].

To date, the e-nose cannot replace other measurement techniques. As it only offers the possibility for comparing one “smell” with another, and it provides no information about the concentration of a given substance. In the other words, it allows for comparative rather than absolute measurements, detecting differences between objects of the same type [9]. However, taking into account its relatively low price, it can be a very comfortable tool for screening tests (for instance, the check about whether there is pollution or not). The objects selected by such screening can be further investigated by more accurate, but more expensive, methods.

Moreover, any interpretation of complex, multidimensional e-nose signals needs a sophisticated approach to data analysis, such as PCA [10] or ANN analysis [11,12]. These methods either allow for finding meaningful information in excessive data sets [13] or are useful, given the highly non-linear nature of dependency among the observed values and the lack of analytical models [14,15].

Recently, the significant broadening of environmental and agricultural applications of the e-nose is noted in all areas where differences and/or changes in a smell of any object occur, such as detecting environmentally hazardous substances and pollutants [16,17,18,19,20], estimating the toxicity of incineration [21], composting [22], rendering [23], checking the quality of wastewater in treatment plants [24,25,26,27,28], detecting fungal contamination of cereal grains [29] and buildings [30], characterizing plant residue decay [31], quantifying odours from livestock and poultry farms [32,33], and monitoring the composition of piggery effluent ponds [14]. Only a few applications of the e-nose in soil science have been reported to date. The available papers concern soil microbial activity [34], discrimination between soil types [35] or the effect of soil tillage history on volatile emissions from crops [36]. Even if a common feeling is that soils smell differs at various moistures, which is particularly evident after rainfall, the literature offers no detailed observations on the moisture effects on soil volatile fingerprints. Some information about this problem is provided by Bastos and Mangan [35] for three soils, two soil water potentials and three temperatures. They used very moist (pF = −0.7 MPa) and very dry (pF = −2.8 MPa) soils highlighting differences in moisture-dependent e-nose signals. This paper was the attempt to measure the e-nose signals for soils of much similar moisture to find the eventual moisture ranges in which the e-nose is applicable in order to differentiate the soil moisture levels. Bastos and Mangan [35] also used very different soil types: a sandy loam, a calcareous clay soil and a volcanic ash, which, by intuition, should also have very different volatile fingerprints. In the research presented in this article it was used the wider set of soil types, which are parallelly more similar in physicochemical properties, in order to check to what extent the soil types are really distinguishable by the e-nose.

To spread the use of the e-nose in systematic methodical work within soil science, a better understanding of the e-nose response in relation to changing soil conditions is needed. It seems that two of the most important factors influencing many soil processes is soil type and water content. Whether these factors influence the smell (the different values obtained by the e-nose) or not, investigations are very important before much wider use of the e-nose in soil sciences. Therefore it is the aim of this work.

## 2. Materials and Methods

Ten different mineral soils, which were collected from across Poland and typical for Central Europe [37], were studied. The disturbed samples were taken from the soil pit [38] of the arable layer (5–20 cm depth). The samples were placed in clean plastic bags and, just after transportation (a maximum of one day), dried to the AD state. Their basic properties, including soil group classification according to the World Reference Base (WRB), are summarized in Table 1.

The soils were stored for about one year in AD conditions, gently crushed in a mortar and passed through a 2 mm sieve. Samples of the sieved soils (75 g) were placed in glass cylinders with a 2.5 dm^3^ capacity (0.9 dm in diameter and 4.0 dm in height). Prior to the experiments, the cylinders were carefully washed, dried at 200 °C and tested with the e-nose to verify the signals’ stability. It was confirmed that e-nose signals, which were measured at the beginning of the experiment, were the same and stable in each of the empty cylinders. 

The first e-nose signals were registered one day after filling the cylinders with AD soils. Then the soils were taken out of the cylinders and moistened with water to obtain homogenous mixtures with the same water content (21.7%), which was equivalent to the value of the average field water capacity for all soils studied. The moistened soils were again placed in the cylinders and uniformly spread on the bottom of the cylinders. Next, the cylinders were covered with a sterile aluminium foil, with a 3 mm hole in its centre. This allowed the soil to dry slowly and retain most of its emissions in the cylinder headspace. The next e-nose signals were registered 1, 7, 8, 15, 22, 44, 71, 100 and 180 days after soil wetting. The soil water content was monitored by weighing all of the cylinders (with the soil inside). Throughout the entire experimental cycle, the cylinders were stored in a dark chamber ventilated with synthetic air at 20 ± 1 °C. The relative humidity (RH) and temperature (T) in the cylinders’ atmosphere were respectively measured using HIH-4000 (Honeywell, Morris Plains, NJ, USA) and DS18B20 (Maxim Integrated, San Jose, CA, USA) sensors. All measurements were replicated thrice.

The e-nose device’s construction was based on eight metal oxide semiconductors (MOS-type gas sensors) manufactured by Figaro (Figaro USA Inc., Arlington Heights, IL, USA): namely, TGS2600-B00, TGS2610-C00, TGS2611-C00, TGS2612-D00, TGS2611-E00, TGS2620-C00, TGS2602-B00 and TGS2610-D00. They are relatively small, with a low power consumption of around 300 mW. Performance of the sensors is based on changes in the electric resistivity (or conductivity) of sensing elements, due to surface chemical reactions between gas molecules and the semiconductor. The intensity of this reaction is proportional to the gas composition and concentration. Each sensor provides a different signal response according to its own sensitivity characteristics, as presented in Table 2.

The applied device was equipped with voltage dividers, which are commonly used as a measurement circuit. The values read by each sensor during the measurement were expressed in ohms (Ω). MOS sensors, applied in the measuring device, were distributed in a polar array and covered with a head, which provided an equal gas flux and stabilized the temperature in the measurement chamber. Before the experiment, the sensors were pre-calibrated with a set of single chemical substances of standard concentrations, which were specific to particular sensors. Signals from all eight implemented sensors constituted a full e-nose array response. The detailed description of the equipment is presented by Guz *et al.* [27,39].

By having a rather small volume of the studied gas sample, the polyamide tube (with a 2 mm inner diameter), which was connected to the e-nose sensor’s chamber, was passed through the aluminium foil hole, arriving at 2 cm above the soil surface. The membrane micro-pump (FM1101 F6V Fürgut GmbH, Tannheim, Germany) sucked out the air from a chamber with 100 cm^3^∙min^−1^ speed, such that, after five minutes of measurement, around 20% of the cylinder atmosphere was replaced by the chamber air. Ten minutes before each series of measurements, the e-nose sensors were flushed with a synthetic air, as well as two minutes before each subsequent measurement. The measurement was conveyed with a 1 Hz reading frequency. For further data expression, the dimensionless relative resistance was determined as an *Rs/Ro* ratio, where *Rs* [kΩ] denoted the average sensor resistance of 15 read-outs from the most stable region at the end of the sample measurements, while *Ro* [kΩ] denoted the average sensor resistance of 15 read-outs from the most stable region at the end of the flushing cycle of the synthetic air.

The PCA [10,41] and ANN [11,42] methods were used to interpret the data. The essential element of PCA is using the existing multidimensional data to create new independent variables (described in the axes as components), which describe the variability of the analysed data set. The newly designed variables have no direct physical meaning and show their percentage contribution in relation to the total covariance of the data set. The PCA enables a reduction in the number of dimensions of the data set. Despite the transformation losing some part of the data, it allows for the results to be presented in a readable form, such as diagrams, so that PCA is a technique of data compression with the possibility of getting back the primary values from the main component space. Axes of the diagrams formed during the analysis represent the main directions of changes that occur in the analysed data, while the measuring data are represented by the vectors showing the directions of the occurring changes. For the obtained data post-processing it was applied PCA based on the covariance matrix due to non-significant differences in the variance of inputs.

Additionally, data processing was supplemented by ANN tests to find out whether volatile fingerprints of a given soil differ enough to distinguish its moisture. For the analysis, a feedforward ANN [42] was used. The ANNs, that are commonly used to analyse signals from an e-nose [13,14,18,43,44,45,46], make it possible to cope with non-linearly separable problems.

**Figure 1:**
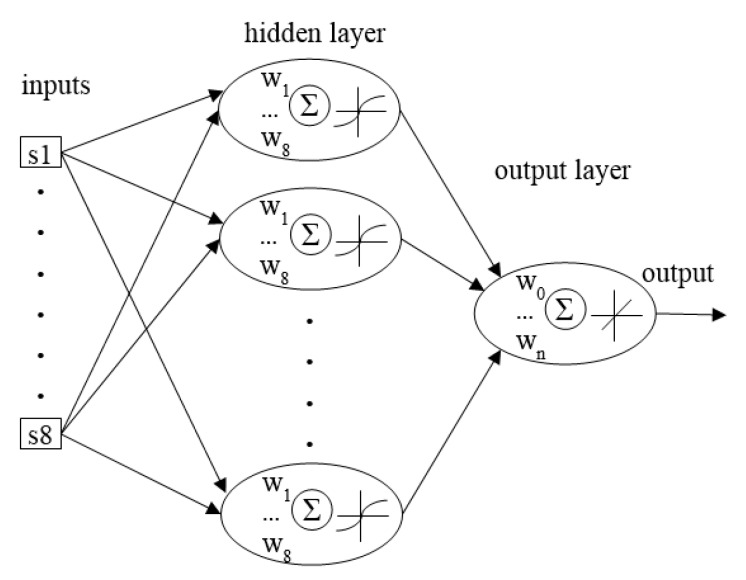
Architecture of the neural network elaborated to analyse soil gas fingerprints.

ANNs are based on the structure of the human brain, in which the neurons create a net of interconnections collecting signals from other neurons and sending a transformed signal to other cells. The essence of the ANNs’ performance is based on the mathematical model describing the principle of information processing. A graphical presentation of the model is presented in Figure 1, wherein the following elements are distinguished: input, weighing, summing up, activating and output. An element, which weighs the input signals, imitates the biological synaptic connection. In the sum of the element ∑, the process of summing up the weighed signals occurs and the *e*-signal is transferred by the activation function. The outcome of this transfer is the output signal, which resembles the signal transferred by the cell axon in the brain.

The architecture of the net consisted of eight inputs, one hidden layer with *n*-neurons and 1 output neurons. The number of hidden neurons *n* was determined according to a general suggestion [47], in which the number of hidden neurons should be less than *2N + 1* and where *N* is the number of inputs (the number of sensors in the present case). The architecture (Figure 1) of the net was determined in order to maximize the generalization ability of a net at its minimal complexity.

For neural network training, the Levenberg-Marquardt learning algorithm was implemented to adjust the weights (w_i_). The total number of data set elements was equal to 4500 records (10 measurement sessions × 10 different samples × 3 replicates × 15 stable readings in each measurement). From the entire data sets, the learning (50%), testing (25%) and validation (25%) subsets were selected randomly. Training subset was used by the network for training while the network was adjusted according to its error. Validation subset was used to determine network generalisation and to stop training when generalisation brought no improvement. Finally, testing subset had no effect on training and so it provided an independent valuation of network performance during and after training procedure. The mean square error (MSE) was used to evaluate the network output error during training. A hyperbolic tangent sigmoid and a linear function were used as transfer functions for hidden and output neurons, respectively. The input data was normalised by linear scaling of a minimum value of 0 and maximum value of 1 [48,49].

The ANNs have been particularly able to analyse multidimensional data, especially if there is no evidently strong relationship between observations. Since an ANN is considered as a “black box”, in order to facilitate the visualization of measurements, many scientists prefer statistical methods, particularly PCA.

## 3. Results

**Figure 2:**
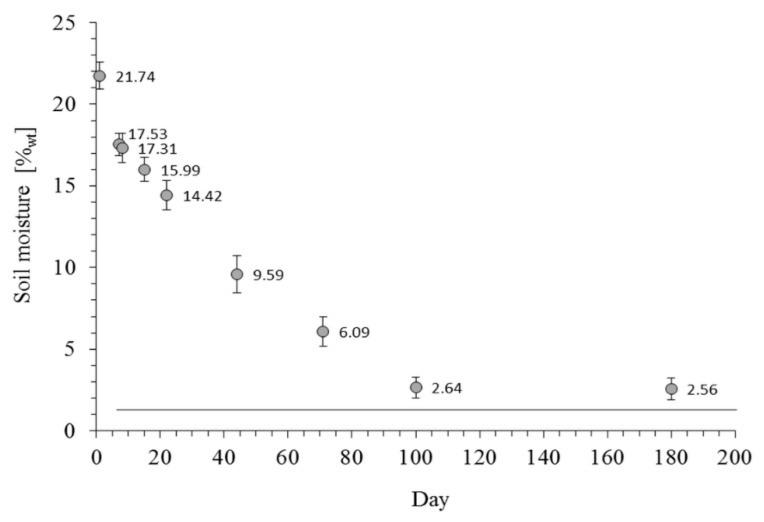
Average values of moisture for all studied soils in the course of drying. Error bars show standard deviations. The line shows the moisture for AD soil.

Changes in average moisture for all soils are presented in Figure 2. For all samples, the moisture was stabilized after the 100th day of the experiment, when it practically reached the AD soil moisture.

**Figure 3:**
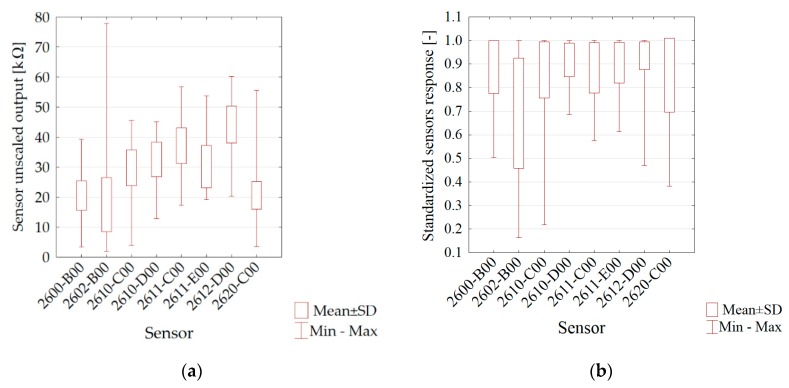
Statistics of sensor outputs variability: (**a**) raw outputs (**b**) scaled outputs.

The similar course of the moisture *vs.* time curves for soils is due to similar initial moistures and drying conditions. Figure 3 presents the basic statistics of the sensor matrix read-outs in the form of a box-and-whisker plot. The ordinates’ axis shows the value of the signals from the particular sensors for all soils samples regarding the phase of the matrix flushed with air. The highest variability was noticed for the 2602-B00 sensor, applied for general air contaminants (with a high sensitivity to VOC and odorous gases), and 2620-C00, applied for alcohol and solvent vapours.

During the whole measurement period before each step it was executed initial 2 min flushing of the sensors with pure air with constant purity class (synthetic air). For that reason for sensors stability evaluation there were used readouts from 15 terminal seconds of sensors flushing. Test results presented in Table 3 were obtained for non-scaled data. The highest standard deviations were observed for the following sensors: 2602-B00 and 2611-C00. With the obtained dependences of standard deviations and the whole readouts variability range during the experiment one may state that the stability of readouts is satisfactory—range from 0.0095 to 0.036.

**Figure 4:**
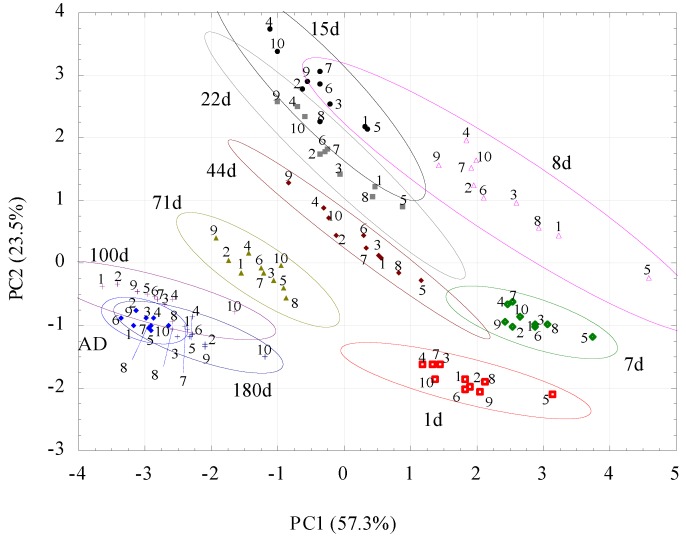
Two-dimensional PCA plots for volatile fingerprints for the 10 studied soils at 10 moistures. The same colours abbreviate the same moistures (AD state, d is days after moistening). The numbers abbreviate soils according to Table 1. The ellipses surround 95% confidence intervals.

Results of PCA of volatile fingerprints of the studied soils are presented in Figure 4. Using PCA it was reduced the eight-dimensional data space (eight e-nose sensors) to a two-dimensional data covariance area with the *x*-axis representing ~57% and the *y*-axis representing ~24% of the whole covariance. The applied method lost ~19% of the information, which is an acceptable level for the conducted test.

Additionally, the ellipses were drawn to fit and enclose the data series. The length of their horizontal and vertical projections onto the *x*- and *y*-axes, respectively, is equal to the *mean ± (range × I)/2*, where the *mean* and *range* of the cluster refer to the PC1 or PC2 variable, while *I* is the value of the coefficient equal 0.95.

The sensors 2602-B00, 2612-D00 and 2611-C00 have higher contribution for the PC1 component and sensors 2610-D00, 2620-C00 and 2610-C00 for the PC2 component (Table 4).

**Figure 5:**
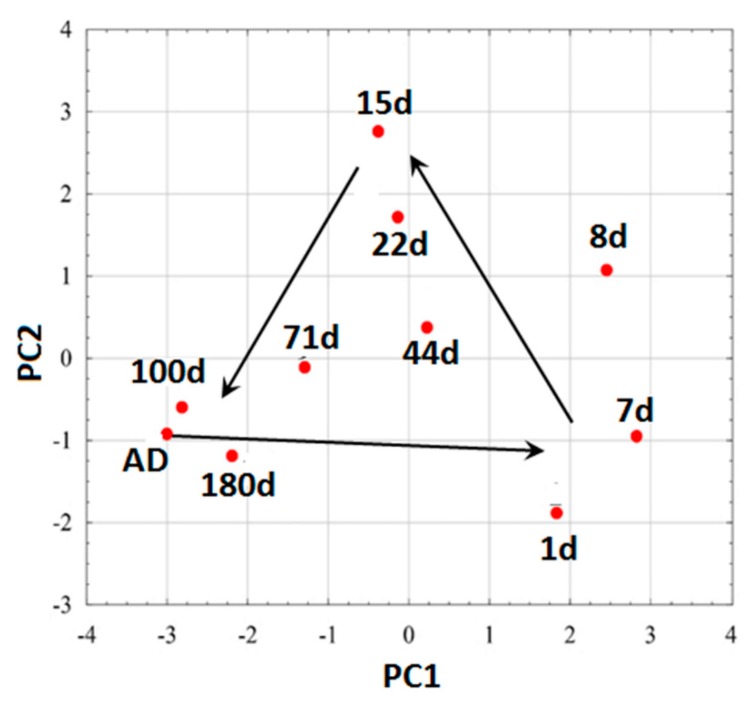
PCA plots of averaged gas-fingerprints for all soils during measurements at various soil moisture.

For all moistures, the PCA results group together on straight lines of similar slopes. The dispersion of the results is the lowest for AD soil, with the largest on the eighth day after moistening. Some trends in the location of points representing soils occur at a given moisture: soils 2, 4 and 9 are most frequently found in the upper left corner, whereas soils 5 and 8 are found in the lower corner. However, to better visualize apparent trends in volatile fingerprints *vs.* soil moisture, Figure 4 was converted to Figure 5, which showed average results for all soils at particular moistures.

One can see that average results form a closed loop beginning with AD soils and closing roughly at the same position. Moistening the soils markedly changed their volatile fingerprints: initially, they are shifted to the right, then upper left before they return to the starting point with lowered moisture. Since PCA analysis differentiated the volatile fingerprints at various moistures, it was checked whether these differences are high enough to be quantified by the elaborated neural networks. Five were used neural networks estimating continuous function due to the possibility to treat particular measuring days as correlated with moisture content of the samples (%_wt_). Three neural networks were selected. Each one was trained after initial random data sequence reorganizing. Additionally there were randomly determined the data sets belonging to training, validation and testing sets. In the Table 5 there are presented the results of MSE error evaluation for the mentioned procedures as well as mean correlation coefficients R between moisture content estimated using network and determined gravimetrically.

The applied ANNs were able to distinguish soil volatile fingerprints measured at various moistures with mean R 0.997 value (Table 5). This confirms that application of artificial neural networks is an effective tool for e-nose signal post-processing.

## 4. Discussion

The research described in this paper presents the possibility of an e-nose application to evaluate the moisture status of several soil types. For data processing there were used the PCA and ANN methods, which enabled to distinguish between different types of soils and their moisture statuses. 

The most clustered results of the first measurement, (AD soils in Figure 3), indicate that the storage of a soil in AD conditions somehow equalizes the influence of the soil origin (type) on the volatile fingerprint. The microbial activity of AD soil is practically zero; however, the microorganisms can still be reactivated [50].

The addition of water to dry soils changed the e-nose signals, suggesting changes in the volatile component composition. By having the same initial water content and similar rate of drying for all soils (a relatively small value of standard error in Figure 2), one can assume that the observed changes in the volatile fingerprints are most probably caused by the cumulative effects of physicochemical and microbiological processes. Initially (one day after moistening), when the soil microorganisms are not yet very active [51,52], it can be expected that the replacement of volatile substances, adsorbed on the surfaces of soil components by water molecules, was the dominant contributor to the e-nose signal.

Next, volatile substances produced by living microorganisms, including products of the decay of soil organic matter, evolve and alter the e-nose response. It is commonly accepted that full microbial activity is achieved between two and seven days after moistening of the dry soil [53,54]. This intensive biological life could shift the PCA results to the upper right and cause a large dispersion of the e-nose signals observed from day eight to 44 of the experiments. Here, the effect of different groups of bacteria growing at various moisture conditions may be pronounced. This is because the drying process increases the heterogeneity of intrinsic soil moisture distribution. Water first evaporates from large soil pores, which become filled with oxygen, giving rise to aerobic bacteria development. From air-filled pores, the easier emission of the volatile substances to the headspace should occur due to much higher diffusion in the gaseous, rather than liquid, phases [55]. Finer pores, which are still filled with water, may contain living anaerobic bacteria populations [56,57]. Therefore, a much wider spectrum of bacterial species is active at the same time. It may be expected that different species of bacteria release different volatile substances into the atmosphere, leading to large variations in e-nose signals and a high dispersion of PCA results. Significantly, smaller dispersion was noted in further soil drying, when the microbial populations became depressed by dry conditions and finally reached a state of dormancy in AD soil. The dispersion of e-nose signals at the 180th day of the experiment is still higher than in the AD soil, despite very similar soil moisture, which may certify that either some microbes still survive in the finest pores, where water and some nutritive substances are still available, or that the composition of soils changed due to the exhaustion in organic components consumed by bacteria.

The differences in PCA plots (Figure 4 and Figure 5) are very promising in the context of recognizing soil moisture status. However, the differences between the soils may have been, in some cases, too large to allow any identification of water content. This problem was checked by ANN analysis. This result is a very good starting point for using the e-nose/ANN combination for further soil investigations.

It is promising that, at a given moisture status, the volatile fingerprints vary between soils. For instance, the location of soils 5 (Mollic Stagnic Fluvisol) and 8 (Haplic Luvisol [Siltic]) is usually at the right, while soils 4 (Leptic Cambisol) and 9 (Leptic Skeletic Dystric Cambisol) are usually at the left side of the PCA plots obtained at the same moisture. This means that it could be possible to distinguish different soil types based on the e-nose signals, provided that their moistures are similar [35]. However, having only one representative from each of the reference soil groups, more studies are necessary to certify this hypothesis.

As compared to Bastos and Mangan [35], results presented in this research for a significantly larger range of soil moistures can provide a stronger base for assessing and characterizing soil moisture by e-nose. However, taking into account that some soils have a similar localization of e-nose signals within the overall data set it may be assumed that further intensive studies on soil type recognition by the e-nose are needed in order to better understanding.

## 5. Conclusions

The general idea behind using the e-nose in soil investigations was embedded in the aim of this work, *i.e.*, the verification whether volatile fingerprints can be interpreted in terms of soil water content. Studies of 10 different soil types at 10 moisture levels showed that the e-nose is a very promising tool.

The combination of the e-nose measurements of volatile fingerprints with PCA, in order to enhance the differences and the ANN for evaluation, satisfactorily recognized water content in soils. The mean square error (MSE) of the best developed network was equal 0.00889, 0.01185 and 0.05011 for training, validation and testing respectively. It also seems that the e-nose may be used as a supporting tool for soil classification. At the same soil moisture levels, the same soil types follow similar locations in PCA plots. However, much more investigation is necessary to prove this statement.

## Figures and Tables

**Table 1:** Basic properties of investigated soils.

No.	WRB Soil Group	Particle Size Group	C_org_ (%) *
1	Brunic Arenosol	Sand	0.86
2	Stagnic Luvisol	Sandy loam	1.19
3	Haplic Cambisol	Sandy loam	0.57
4	Leptic Cambisol	Silt loam	1.08
5	Mollic Stagnic Fluvisol	Silt loam	1.14
6	Stagnic Phaeozem (Siltic)	Silt	1.97
7	Haplic Chernozem (Siltic)	Silt loam	1.11
8	Haplic Luvisol (Siltic)	Silt	1.06
9	Leptic Skeletic Dystric Cambisol	Silt loam	0.90
10	Haplic Fluvisol (Clayic)	Silt	1.86

C_org_*—the content of organic carbon.

**Table 2:** Overview of the gas sensors implemented in the e-nose [40].

Sensor Type	Description	Detection Range	Sensitivity
TGS2600-B00	general air contaminants, hydrogen, ethanol, *etc.*	1–30 ppm of hydrogen	0.3–0.6 for $\frac{Rs\left(10\;ppm\;H_{2}\right)}{Rs\left(air\right)}$
TGS2602-B00	air contaminants, toluene, VOCs, ammonia, hydrogen disulfide	1–30 ppm of ethanol	0.08–0.5 for $\frac{Rs\left(10\;ppm\;Ethanol\right)}{Rs\left(air\right)}$
TGS2610-C00	butane, LP gas	500–10,000 ppm	0.56 ± 0.06 for $\frac{Rs\;\left(3000\;ppm\right)}{Rs\;\left(1000\;ppm\right)}$
TGS2610-D00	butane, LP gas (carbon filter)	500–10,000 ppm	0.56 ± 0.06 for $\frac{Rs\;\left(3000\;ppm\right)}{Rs\;\left(1000\;ppm\right)}$
TGS2611-C00	methane, natural gas	500–10,000 ppm	0.6 ± 0.06 for $\frac{Rs\left(9000\;ppm\right)}{Rs\left(3000\;ppm\right)}$
TGS2611-E00	methane, natural gas (carbon filter)	500–10,000 ppm	0.6 ± 0.06 for $\frac{Rs\left(9000\;ppm\right)}{Rs\left(3000\;ppm\right)}$
TGS2612-D00	methane, propane, iso-butane, solvent vapors	1%–25% LEL *	0.5–0.65 for $\frac{Rs\left(9000\;ppm\right)}{Rs\left(3000\;ppm\right)}$
TGS2620-C00	alcohol, solvent vapors, carbon oxide, hydrogen	50–5000 ppm	0.3–0.5 for $\frac{Rs\left(300\;ppm\right)}{Rs\left(50\;ppm\right)}$

* VOC—volatile organic compounds, LEL—lower explosive limit.

**Table 3:** Stability of sensors during a flushing cycle.

Variable	Mean (kΩ)	Min (kΩ)	Max (kΩ)	SD	SD/Variability
2600-B00	21.54036	20.39690	22.25086	0.453896	0.01267
2602-B00	44.42514	38.94425	47.85411	2.232958	0.02946
2610-C00	33.41887	31.82044	34.55444	0.654846	0.01558
2610-D00	40.69828	38.78311	41.76918	0.566877	0.01756
2611-C00	46.59227	44.62462	51.32284	1.440915	0.03627
2611-E00	41.10873	39.58662	42.15165	0.441327	0.01276
2612-D00	55.10230	53.02001	56.51318	0.664912	0.01664
2620-C00	22.67450	21.48789	23.51278	0.493596	0.00950

**Table 4:** Variable contribution to PCA.

Variable	2600-B00	2602-B00	2610-C00	2610-D00	2611-C00	2611-E00	2612-D00	2620-C00
PC1	0.007752	0.202078	0.143791	0.060383	0.117228	0.19586	0.197858	0.075051
PC2	0.004066	0.004421	0.190849	0.362531	0.011935	0.058742	0.043401	0.324054

**Table 5:** Results of five neural networks detecting soil moisture status—data for all soils of a given moisture were treated as a single data set.

Net ID	Training		Validation		Testing	
	MSE	R	MSE	R	MSE	R
1	0.04122	0.9995	0.06046	0.9993	0.03647	0.9960
2	0.00889	0.9999	0.01185	0.9998	0.05011	0.9994
2	0.02807	0.9997	0.05587	0.9994	0.07800	0.9991
4	0.05226	0.9994	0.06437	0.9993	0.06812	0.9926
5	0.05044	0.9994	0.17490	0.9981	0.05910	0.9993
